# A *One Health* perspective on the issue of the antibiotic resistance

**DOI:** 10.1051/parasite/2021006

**Published:** 2021-03-19

**Authors:** Estera Badau

**Affiliations:** Laboratoire CIMEOS EA4177, Université de Bourgogne 4 boulevard Gabriel 21000 Dijon France

**Keywords:** One Health, Antibiotic resistance, Discourse analysis, Formula, Action program

## Abstract

For a few years now, the *One Health* concept has appeared to go hand in hand with the issue of antibiotic resistance as the most comprehensive and global solution. As part of a study comparing the publicization process of the links between antibiotic resistance and food in France and in the United States, this paper retraces the *One Health* concept’s trajectory in terms of significations and (re)definitions, according to the actors adopting this approach as a viable solution. Furthermore, this paper questions the concept’s take over impact in antibiotic resistance reframing as well as its expansion in terms of functioning and applicability. Within social sciences research, interest in the issue of antibiotic resistance and the *One Health* approach has largely been established in recent years by a growing number of studies examining its different and multiple aspects. The specificity of this research lies in its two different levels of questioning the *One Health* concept. Firstly, the concept seems to be referred to by various *formulas*, from its oldest form, *One Medicine*-1984, to *One World, One Health*. Secondly, the concept is being redefined as links between a plurality of domains are recognized (human health, animal health, the environment, and food), following the emergence of international health and food crises and as their multi-level consequences are being addressed by various stakeholders, including public authorities, political leaders, and economic actors.

## Abstract (fr)

Depuis quelques années, le concept d’une *seule santé* semble aller de pair avec le problème de l’antibiorésistance en tant que solution nécessaire la plus appropriée et la plus globale. Issu d’une étude comparant le processus de publicisation des liens entre l’antibiorésistance et l’alimentation en France et aux États-Unis, cet article retrace la trajectoire du concept *One Health* en termes de significations et de (re)définitions selon les acteurs ayant recours à cette approche comme solution viable. De plus, l’article s’intéresse à l’impact du concept dans le recadrage du problème de l’antibiorésistance ainsi qu’à son expansion en termes de fonctionnement et d’applicabilité. Dans le cadre de la recherche en sciences sociales, l’intérêt pour la question de la résistance aux antibiotiques et l’approche *One Health* a été largement établi au cours des dernières années par un nombre croissant d’études examinant ses différents et multiples aspects. La particularité de cette recherche repose sur ses deux niveaux différents d’étude du concept *One Health*. Tout d’abord, le concept semble circuler à travers différentes *formules*, de sa forme la plus ancienne, *One Medicine*-1984, à *One World, One Health*. Deuxièmement, le concept est en train d’être redéfini à mesure que les liens entre une pluralité de domaines sont reconnus (santé humaine, santé animale, environnement, alimentation), à la suite de l’émergence de crises sanitaires et alimentaires internationales et au fur et à mesure que la prise de conscience de leurs conséquences à plusieurs niveaux est reconnue et abordée par divers acteurs (autorités publiques, dirigeants politiques, acteurs économiques).

## Introduction

In 2016, the 71st session of the United Nations General Assembly focusing on – sustainable development goals, had the issue of antibiotic resistance (i.e. the ability of bacteria to develop forms of resistance to antibiotics [2]) on its agenda. While this was the fourth time in its history that the United Nations has placed a health issue on its agenda – the other three being HIV/AIDS, non-communicable diseases, and Ebola – this marked a turning point in the process of publicizing the antibiotic resistance problem and taking action. Within public problem studies [3–5, 15, 27–30], the processes through which a *problematic situation* [15] is publicized, and becomes a public problem include phases such as definition and demand for a solution, and political recognition in order to develop and implement public policies. As a result of these phases, the solutions are often developed as *action programs* [61] in order to resolve the problem. Proof of the political awareness and recognition of the links between the various fields involved in antibiotic resistance, as well as of its economic and societal consequences, the session resulted in a declaration of international political commitment. This commitment made by world leaders underlines the need for multi-sectorial cooperation and the implementation of national plans in accordance with the objectives of the Global Plan launched in 2015 by the three international agencies (WHO, OIE and FAO) following the *One Health* approach, i.e. the recognition of links between human health, animal health, and the environment [55].

Seen through the prism of public problem analysis, this moment reveals two important aspects. The first aspect is related to the political recognition phase which implies the search for solutions [4]. This search has led to the development of new concepts (or the redefinition of existing concepts – in this case *One Health*), concepts designed to provide solutions. The second aspect is related to the actors doing this work (and their specific features and interest). As a result, different *formulas* (and different definitions) designing the *One Health* solution have emerged. This work investigates this process in order to understand and clarify what *One Health* represents for the main actors involved in defining the solutions to the antibiotic resistance issue.

### Concept development in the light of the emergence global crises

The study of new concept development in relation to the significant health crises and pandemics of recent decades has led to an increasingly broad participation of stakeholders in the search for solutions. As previously argued [4], since the late 1980s, the increasing number of health and food crises [12], and their global spread has revealed the need to mobilize a plurality of actors from different sectors in order to manage and control the consequences. Therefore, the search for solutions has led to the development of new concepts or the redefinition of existing concepts designed to provide solutions. The various crises, such as the threat of dioxin in food, mad cow disease (bovine spongiform encephalopathy; BSE), or avian influenza are just a few examples that have demonstrated the need for international governance, and have resulted in the development of various concepts. Particularly, the management of the Belgian dioxin crisis (1999) led to the emergence of the concept of *food traceability* [7], and France’s Infected blood scandal (1991) led to the emergence of the concept of *health safety* [9] in the health field. In the context of the mad cow disease crisis mainly in the UK (1980s and 1990s), the concept of *food safety* was redefined. Through national and international collaborations, new institutions were created: AFSSA in 1999 – the French agency for food safety and its European equivalent, EFSA – the European Food Safety Authority, in 2002 [7].

Looking at antibiotic resistance through the *One Health* perspective requires us to retrace the history and the roots of this concept, as well as to explain the context in which it has been adopted by health and political actors as an approach to combat antibiotic resistance.

## Materials and methods

### Materials

The analysis is based on a compiled but non-exhaustive corpus that includes different types of documents: reports, press releases, and commitment statements, issued by health, political, or private actors, and linked to a series of events related to antibiotic resistance, such as: the 2016 World Economic Forum in Davos, the “Review on antimicrobial resistance” group’s publications, the 2016 G7 and G20 meetings, and the UN 71st meeting (see the Appendix for the complete titles of the documents). These events took place on different *public scenes* [15], where various types of actors are involved. Therefore, there are different consequences of defining the problem and the solutions to be developed and applied. The selection criteria for these documents published in 2016 are connected to public problem studies. In fact, within public problem studies [3, 4, 15, 27–30], the processes through which a problematic situation is publicized and becomes a public problem include phases such as political recognition in order to develop and implement public policies. The momentum that the antibiotic resistance problem achieved in 2016 around the series of events held by the international health organizations, but also by political actors, revealed the political recognition of a *One Health* approach needed for antibiotic resistance. This political recognition, a phase of development of a public problem, requires us to look into the specific discourses held by the actors involved, whether they are strictly political actors, international health organizations, or private actors. Therefore, we investigated at least one document published by each type of actor. The objective was not to establish an exhaustive list of documents and reports, but to see how the concept is defined by these actors at this particular moment, since these definitions might reveal (re)frames of both the *One Health* concept as a solution as well as the antibiotic resistance problem. However, in order to understand and analyze the moment’s significations and positioning of the actors, a retrospective study in regard to the *One Health* concept is required. Hence, we looked into the concept’s trajectory.

### Methods

Following a historical approach regarding the concept’s evolution in terms of definition and signification, this paper relies on a discourse analysis method. As discourse analysis is a broad field of research, the qualitative method follows the semantic-pragmatic approach and is based on several concepts. The plurality of approaches to discourse analysis from different intellectual currents raises questions about the definition of what discourse is. Our work considers discourse, following Dominique Maingueneau, as a “set of strategies of a subject whose product will be a construction characterized by actors, objects, properties, and events on which it takes place” [44]. Indeed, the things highlighted by this definition concern the importance of the conditions of producing a discourse, of the context, and the actors in the construction of the meaning of a discourse. To this, we add the *formula* definition developed by Alice Krieg-Planque and explained as “the set of formulations which, because of their uses at a given time and in a given public space, crystallize political and social issues that these expressions contribute at the same time building.” [40] Applied to the study of public problems and concepts development, these observations make it possible to observe the role played by the different groups of actors taking ownership of the problem in the emergence of names of the problem and its solutions. These tools also allow us to analyze the various positions taken by different actors in the circulation process of names of a problem in the public space. As Jocelyne Arquembourg’s work shows [3], these considerations allow the study of the process through which the multiplicity of the aspects related to a problem are grouped under a single name, the name of the problem. Furthermore, based on Joseph Gusfield’s proposals [30] and Jean Widmer [61], the antibiotic resistance problem can be analyzed as the subject of various action programs [5], implementing several types of solutions. For example, while the pharmaceutical industry has called on governments to fund research into new antibiotics, agri-food industry stakeholders have participated in the development of the “antibiotic-free” concept, which circulates under a plurality of formulas [4]. To sum up, as stated by Joseph Gusfield, “facts take on significance through the processes by which they are selected for attention, named, classified and given a relationship to each other. […] At the same time, we need to take care not to separate the study of meanings from the study of their historical and institutional settings” [29]. This method will therefore allow us not only to understand the process through which new concepts emerged (or how old ones are redefined), but also to analyze the part played in this process by the actors, furthermore, their positioning and the role played in the development of solutions.

## Results

### What does *One Health* represent? From *One Medicine* to *One Health*: ownership and (re)definitions

Within social sciences research, interest in the *One Health* approach (applied to various health problems and global crises) has been largely established in recent years by a growing number of studies examining its different and multiple aspects [13, 16, 34, 41, 64]. The *One Health* concept finds its origins in the old *One Medicine* formula, invented by veterinary epidemiologist Calvin Schwabe [59]. It is closely linked to the zoonosis concept conceived by the German veterinarian Rudolf Virchow in the 19th century, to name infectious diseases that can be transmitted between humans and animals. In view of these considerations, Schwabe introduced the concept of *One Medicine* as a call for a unified approach between human and animal medicine to combat zoonoses [59]. Angela Cassidy’s recent work [13] shows that since the 2000s, the concept has been taken up by many international agencies and non-governmental organizations, as well as veterinary associations, as a call for the integration of the human and animal health fields, while adding the field of the environment, in response to the growing importance of health threats raised by several global problems including global warming, food insecurity, and the emergence of new infectious diseases. As the author points out, the call for the integration of these three areas into a common vision in the early 2000s reflected the need for collaboration in medical research, public policy, and clinical practice. At this stage, we can see a preliminary redefinition of the concept of *One Medicine*, a definition that at the beginning of the 20th century also included the environment alongside human and animal health, and which subsequently extended from medical research and clinical practices to the implementation of public policies.

Following this movement, which appeared in the context of an explosion of diseases showing various links between these three fields, such as West Nile Virus disease, severe acute respiratory syndrome (SARS), mad cow disease, and avian influenza, in 2004 the concept was adopted by the non-governmental association Wildlife Conservation Society, which organized in collaboration with Rockefeller University in New York, the first international conference “One World, One Health: Building Interdisciplinary Bridges to Health in a Globalized World”. Under this new formula, *One World, One Health*, which aimed to rename the *One Medicine* concept, led to the publication of a preliminary international guide, “The Manhattan Principles” [62, 63]. This guide contained twelve recommendations and calls for the development of a holistic approach to prevent, control and monitor epidemics and epizootics in order to maintain ecosystem integrity and biodiversity for the benefit of humans and animals. At this stage, two important aspects can be observed in the development of the *One Health* concept. On the one hand, the emergence of a new formula to name it (*One World, One Health*), while emphasizing the importance of taking the environment into account. On the other, the role played by recent events, including the four zoonotic epidemics: West Nile Virus infection, SARS, mad cow disease, and avian influenza. All these advances have also made it possible to observe that the awareness of the links between the three fields, as well as the management and control difficulties raised by the consequences of these epidemics, have led to a redefinition of the concept by the scientific community under the coordination of the non-governmental organization.

From 2007, simultaneously with the movement initiated by the non-governmental organization, collaborations began between the presidents of the American Medical Association (AMA – Ron Davis) and the American Veterinary Medical Association (AVMA – Roger Mahr). During an interview for our PhD research, Laura Kahn, a physician and researcher at Princeton University and carrier of the concept *One Health*, explained that it is by consulting policies related to disease governance that she realized the lack of collaboration between human and animal health actors in two cases that emerged in the early 2000s: in particular West Nile Virus and bioterrorism (anthrax attacks). She pointed out that, looking at the scientific literature on the two subjects, no study showed that they are zoonotic diseases and that, at the same time, no infrastructure facilitated exchange and collaboration between human and animal health stakeholders. All this research led her to publish the article “Confronting Zoonoses: Linking Human and Veterinary Medicine” in 2006 [38], which received many responses from veterinarians, but no response from the medical community.

It is thanks to the publication of this article that a strong collaboration between veterinarian Bruce Kaplan and the two presidents of the medical and veterinary associations, Ron Davis and Roger Mahr respectively, was born. Laura Khan emphasizes the open-mindedness of Ron Davis, the AMA President, and the fact that he was working on issues related to obesity in humans and dogs. As a result, these aspects have facilitated the dialogue with the medical community. However, as Kahn explains, the term *One Medicine*, as coined by Schwabe, could not have been accepted by physicians because of various misgivings linked to the historical dichotomy between human and animal medicine. In order to attract the interest of their medical doctor colleagues, Davis suggested that Kahn could write a resolution that should be put to a vote. The resolution was accepted on condition that the term *One Medicine* be replaced by the term *One Health*. After Ron Davis’s death a few years later, there was less support from the medical community. However, collaborations with Kaplan led to the creation of the *One Health Initiative* platform, an initiative that helped to develop a new formula: *One World, One Medicine, One Health* [39]. In parallel with this platform, which is intended to bring together the human and animal health scientific community, the American Veterinary Medical Association, in collaboration with the American Public Health Association, founded The *One Health Commission* in 2009 following the publication in 2008 of the report by the One Health Initiative Task Force [1].

Simultaneously with these steps, another major event that took place in 2008 affected the development of the *One Health* concept. This was the pandemic caused by the emergence of the H5N1 virus. In response to the crisis associated with the emergence of H5N1, the three international health agencies, WHO, OIE and FAO, in collaboration with the United Nations International Children’s Emergency Fund (UNICEF), the United Nations System Influenza Coordination (UNSIC), and the World Bank, jointly approved a framework under the aegis of *One World, One Health*, while borrowing the definition formulated by the American Veterinary Medical Association, according to which the approach includes: “the collaborative efforts of multiple disciplines working locally, nationally and globally to attain optimal health for people, animals and our environment” [22].

This new episode, part of the development of the *One Health* concept, is similar to the previous ones. This similarity is reflected in the exact adoption and appropriation of the same concept by several groups of actors following the emergence of crises that link human and animal health, and the environment. Nevertheless, a major difference appeared and is related to the characteristics of groups of actors who take ownership of the concept. As Aline Leboeuf’s report states [41], at this stage, development of the *One Health* concept had given rise to two levels: one that reflected an international governance policy, and the other that showed its characteristics in a context of academic research. However, it is essential to note the multitude of formulas under which the concept has circulated so far. Furthermore, the sociologist Yu-Ju Chien [16] analyzed the appropriation of the concept by international health agencies during the avian flu crisis, while emphasizing the creation of a new cognitive framework. This governance model thus reflected a new policy under the formula *One World, One Health*. At the same time, under this *One World, One Health* governance framework, the legitimacy and the influence of these international agencies with regards to crisis framing and management were underlined.

Following these events, in 2010, the three international health agencies, WHO, OIE and FAO [23], published a new agreement on the concept, while affirming the need for international collaboration in order to develop solutions and measures to control health risks attributable to zoonoses. The document is particularly valuable as it helps redefine the *One Health* concept, which also includes the food sector, but the global nature of the concept remains of paramount importance:

“A world capable of preventing, detecting, containing, eliminating, and responding to animal and public health risks attributable to zoonoses and animal diseases **with an impact on food security** through multi-sectorial cooperation and strong partnerships”.

This redefinition of the concept has two consequences. The first consequence is the redefinition of the very meaning of the concept, since the “food sector” is recognized as part to be taken into consideration along with human health, animal health and environment. The second consequence is related to the actors’ status. As this project was carried out by the reference international health agencies, this redefinition of the concept was supposed to be followed by the national institutions. However, it is important to remember that this is not a regulation with coercive characteristics, and importantly there are various factors to be considered when an international framework is to be implemented at the national level. For instance, looking at the example of France, the first document revealing the position of the French public institutions on the concept was published in 2011 [48]. The definition adopted by the French authorities shows yet another expansion of the concept by adding the economic and social dimensions to the common understanding of the fields of human and animal health, the environment, and the food sector.

Thus, to summarize, the aspects highlighted so far demonstrate the different steps that have enabled the *One Medicine* research concept to be defined as a concept of international political governance following a *One Health* approach, and including several redefinitions according to the categories of actors who have taken ownership of this concept. At the same time, this journey reveals the circulation of several formulas under which the concept has been taken up and redefined. An important aspect that the development of the *One Health* concept reveals is that the transition from its conceptualization at the research stage to its transformation into an international governance mechanism for pandemics, epidemics, and epizootics has been driven by the occurrence of certain events that have resulted in emerging global health crises, revealing the links between human health, animal health, the environment, and food production in a context of accelerating globalization. The recognition of these links by the health actors in the fields concerned and the appropriation of the *One Health* concept have therefore generated two changes in the development of this concept. On the one hand, a redefinition of its meaning, and on the other, at the level of its functioning, the path towards an international governance system. The implicit question raised by these aspects is linked to the emerging tensions between frameworks and governance models at a global or international scale, and the governance and implementation of policies at the national level.

Table 1 summarizes these phases of concept development, with an emphasis on the evolution of the formulas under which it circulates, as well as the evolution of its definition. As a result, heir to the old *One Medicine* concept conceived at the end of the 20th century following the emergence of the first zoonosis and calling for the unification of human and animal medicine, *One Health* now includes the recognition of the links that are being forged between the two sectors of health, human and animal, the environment and food, while advocating a new model for international governance of global health issues.

**Table 1:** The trajectory of *One Health*: formulas and definitions.

Date	Formula	Actor	Definition	Events and causes	Implications
1984	One Medicine	Calvin Schwabe	Call to unify human and animal health	First zoonosis	Research concept
2004	One World, One Health	Wildlife Conservation Society	Human health, animal health, the environment	Global warming, West Nile Virus, SARS, BSE	Recommendation guide: The Manhattan Principles
2006	One World, One Medicine, One Health	Laura Khan, Bruce Kaplan, Roger Mahr (AMVA), Ron Davis (AMA)	Human and animal health, the environment	West Nile Virus, bioterrorism (anthrax)	Platform: One Health Initiative
2007–2009	One Health Commission	AMA and AMVA	Human and animal health, the environment		One Health Initiative Task Report
2008	One World, One Health	WHO, FAO, OIE, UNICEF, UNSIC, World Bank	Local, national and international collaborations between the three areas	H5N1	Governance system for pandemics, epidemics and epizootics
2010	One Health	WHO, OIE, FAO	Human and animal health, the environment, food safety	Integrated approach of health following the globalization of health risks	A concept tripartite note
2011	One Health/Une seule santé	France: Ministry of Foreign and European Affairs	Human and animal health, the environment, the food sector, economic and social dimensions	Increased circulation of infectious agents and risks of pandemics (avian flu, H1N1 flu, SARS)	National ownership of the concept

### Antibiotic resistance through *One Health*: concept ownership and reframing a problem

#### European and international health actors’ recognition and requalification of the problem

The recent adoption of the *One Health* concept as a necessary approach to developing and implementing solutions to the antibiotic resistance problem is rooted in the process of awareness of the links between human and animal health, the environment, and the food production sector, while taking into account the economic and societal consequences they may have. However, it is important to highlight that the scientific acknowledgement of the links between antibiotic resistance and food has not necessarily been followed by political recognition or public policies implementation. Also, as previously argued [4], in different countries around the world, political recognition and public policy implementation did not take place at the same time. For instance, Laura Kahn’s work shows that some of the “Swann report” recommendations (Committee in the United Kingdom) [37] were first implemented in Sweden. The events related to the “avoparcin crisis” and the European ban on growth promoters have also been analyzed by Setbon and taken into account [4]. What this paper examined was the history of adoption of the *One Health* approach as a solution to the antibiotic resistance problem. On one level, there has been an important gap between scientific awareness [8, 10, 43, 45] and political recognition of the links between the various fields concerned by antibiotic resistance. We also need to consider the history of the *One Health* concept, as presented in the previous section. On another level, there has also been a gap between recognizing the different fields concerned by antibiotic resistance and the actual adoption of the *One Health* approach. To analyze what the *One Health* approach means in the context of antibiotic resistance, we must not only have these historical elements in mind, but also look closely at the actors adopting this approach and with what definition. In this regard, the choice of the events of 2016 related to the antibiotic resistance problem is essential to this paper, as the moment represents not only the international health agencies adoption and reinforcement of the *One Health* approach, but also the engagement of international political leaders.

For instance, in the United States, the first article that proves that there are links between the use of antibiotics in animal feed and bacterial changes in the intestinal flora of farmers dates back to 1976 [42]. In contrast, the first WHO report highlighting the need for a *One Health* approach to antibiotic resistance was published in 2014 [54]. What seems even more important to note is the fact that international political leaders have only recently become involved in the fight against this problem. It was in 2016 that this problem was put on the international political agenda by Jim O’Neill. The publication of the report he coordinated highlights the economic and social consequences of the problem (i.e. 10 million deaths each year worldwide, and USD 100 trillion loss in global production in 2050 if nothing is done) [56]. This significant gap between scientific awareness and political recognition also explains the very low media coverage of the concept (almost non-existent during the period 1980–2016) in the French national daily press. Finally, it is useful to mention that, at least in the American case, the steps taken by non-governmental organizations, such as Pew Charitable Trusts (since 2015) followed by the Consumer Union in the same year, are aimed at the need to adopt the One Health approach, without however naming it as such [4].

Thus, the first noteworthy document highlighting the need for a *One Health* approach to antibiotic resistance that we analyzed dates back to 2014. This is the publication of the WHO report on microbial resistance surveillance [54]. This publication was produced in collaboration with the other two international health agencies, the OIE – its animal health counterpart, and the FAO – the Food and Agriculture Organization of the United Nations. The report includes a definition of the *One Health* concept that covers human and animal health, as well as food safety. Admittedly, the definition is not new, but its application to antibiotic resistance shows the emergence of a phase of re-qualification of the problem, which is reflected in the recognition by health stakeholders of the links between the use of antibiotics in human health and animal husbandry, as well as the transmission of resistant bacteria through the food chain. This is presented in the report as follows:

“WHO, FAO and OIE have established a formal tripartite alliance to enhance global coordination and to promote intersectoral collaboration between the public health and animal health sectors as well as in food safety (under the “One Health” approach). The FAO/OIE/WHO Tripartite has identified AMR as one of the three priority topics for joint actions”.

This first report was followed by multiple initiatives organized by international and European health actors. In January 2015, the three European health agencies, the European Centre for Disease Prevention and Control (ECDC), the European Food Safety Authority (EFSA), and the European Medicines Agency (EMA), published their first report based on an integrated analysis of the consumption of antimicrobial agents and the emergence of resistant bacteria in humans and food-producing animals. While the report does not mention the need for a *One Health* approach, it is one of the first examples of evidence that the links between the multiple areas involved in the problem of antimicrobial resistance were being recognized, being in fact commissioned in 2012 by the European Commission. Despite the methodological difficulties in analyzing the data and creating reference indicators, the results based on the data collected in 2011 and 2012 show positive associations between the use of certain classes of the latest generation antibiotics (cephalosporin and fluoroquinolones) and the emergence of resistance to *Escherichia coli* bacteria from humans and from food-producing animals.

The report was followed in 2015 by the launch of the first global plan to combat antimicrobial resistance by the WHO [55], which is divided into five objectives to: i) improve awareness and understanding of the problem in communication, education and training; ii) strengthen knowledge and data by increasing surveillance and relying on research; iii) reduce the recurrence of infections by taking health and preventive measures; iv) optimize antimicrobial use in human and animal health; and v) develop cost-effective procedures for investment in new medicines, but also diagnostic tools, vaccines and other alternatives. As explained by Dr. Margaret Chan, former Director of the WHO, this action plan highlights the need for a *One Health* approach and adopts a definition of the concept that brings together at the same level human and animal medicine, agriculture, the financial sector, the environment, and the consumer. The following excerpt focuses on this redefinition of the concept, which also includes food production and consumers:

“This action plan underscores the need for an effective “one health” approach involving coordination among numerous international sectors and actors, including human and veterinary medicine, agriculture, finance, environment, and well-informed consumers. The action plan recognizes and addresses both the variable resources nations have to combat antimicrobial resistance and the economic factors that discourage the development of replacement products by the pharmaceutical industry”.

The WHO publications were followed by OIE initiatives and resolutions highlighting the need for “prudent use” of antimicrobials [52, 53]. Various elements are extremely important here as OIE’s definition of the issue includes not only antibiotics but also antimicrobials; the definition of the problem also points out use in plants, the solutions focus on “responsible and prudent use”, animal welfare is taken into account, and finally the *One Health* definition is understood as “a coordinated, focused, multi-sectorial and multinational effort”:

“AMR refers to microorganisms, such as bacteria, viruses, fungi and parasites, which have acquired resistance to antimicrobial treatment. AMR may occur naturally as organisms adapt to their environments. However, overuse and misuse of antimicrobial agents in the human, animal and plant sectors has dramatically accelerated the emergence of AMR. Consequently, minimizing the emergence and spread of AMR requires a coordinated, focused multi-sectorial and multinational effort. […] Animal health and welfare depend on the availability, effectiveness, and appropriate use of quality veterinary medicines, including antimicrobials. To continue to progress in disease control management and in improving animal welfare, we as international, regional, national and local animal sector leaders, need to encourage and achieve a sustainable change in behavior so that antimicrobial use in animals closely respects the OIE international standards on responsible and prudent use” [52].

In this way, the OIE *One Health* strategy focusses on the need for surveillance and international standards, through its four main objectives: i) improve awareness and understanding, ii) strengthen knowledge through surveillance and research; iii) support good governance and capacity building; and iv) encourage implementation of international standards. Finally it recommends that “the OIE Strategy on antimicrobials be implemented through a stepwise approach, in close cooperation with WHO and FAO through a *One Health* approach as well as with other concerned partners and stakeholders, and that the OIE further promotes intersectorial cooperation, coordination and interaction at the regional and national levels [53]. The important aspect to note here is that although their discourses highlight international collaboration, when looking closely at the problem’s definition by each agency, discrepancies appear, suggesting competition regarding the ownership of the problem, which subsequently affects the definition of solutions.

#### Political recognition and engagement

These events were followed by the previously mentioned publication in May 2016 of the final report led by Jim O’Neill, which, as we explained, placed the issue on the political agenda of the G7 and G20 international summits. A short parenthesis is required here in order to point out that this final report was preceded by several other publications related to AMR aspects, such as the need for international cooperation between governments and industry in funding research for new antibiotics. Hence, the movement of recognition and commitment was intensified by the emergence of other categories of actors who would take ownership of the problem, such as political leaders and the pharmaceutical industry. As an example, we can cite the statement issued at the annual economic forum held in Davos in 2016 by a group of pharmaceutical, biotechnology and diagnostic industries. Their request to the public authorities concerned the need for funding and public-private partnerships in the search for new antibiotics and other diagnostic tools. In this case, the resulting action program (re)frames the AMR issue from a pharmaceutical economic point of view, while maintaining the need for a *One Health* approach. The declaration attests to their commitment to the project aimed at solving this problem through a *One Health* approach that supports the need for antibiotic use qualified as “responsible” in livestock. The signatories of this declaration clarified their claims as follows:

“We support measures to reduce environmental pollution from antibiotics, along with a ‘one health’ approach towards prudent and responsible use, including a global reduction of unnecessary antibiotic use in livestock, and we applaud moves from major food groups to work towards this goal [58]”.

Regarding the political recognition phase, the declaration from the G7 summit in 2016 highlights two things. Firstly, it was the first time that world political leaders recognized the need to implement a *One Health* approach to combat antimicrobial resistance. Secondly, placing the problem on the global political agenda attests to political recognition of the links between human and animal health, agriculture, food and the environment, while underlining the need for investment funds for research on the problem:

“We commit to make collective efforts for strengthening and actively implementing a multi-sectoral One Health Approach, taking into account the sectors including human and animal health, agriculture, food and the environment. We particularly endeavor to preserve effectiveness of antimicrobials, including by preserving existing antibiotics, to strengthen the inter-sectoral surveillance in all sectors, and to improve access to effective antimicrobials through accelerated support in cooperation with other countries and private sector partners. Recognizing the need to address market failure in which pharmaceutical companies are not producing new diagnostics and drugs required to combat infectious diseases in the face of AMR, we also commit to consider potential for new incentives to promote R&D on AMR and call on the international community to take further action [32]”.

While at the G20 Summit the problem was less central, participants nevertheless underlined their position of support for the efforts of international agencies. In addition, the event was also the subject of a first published report on resistant infections by the World Bank. While describing the problem as a threat to our economic future, and emphasizing that its reduction is a public good, the report defends the imperative of a *One Health* approach. As the following excerpt highlights, the report denounces the lack of collaboration between human and animal health stakeholders:

“Increased global cooperation is essential as AMR containment is a global public good. It will require coordinated efforts to monitor, regulate, and reduce the use of antibiotics and other antimicrobials. It will also require efficient arrangements for adequate and predictable financing of capacities for AMR containment in low- and middle-income countries. [...] The links with veterinary public health have not been adequately addressed to date. It has a substantial interface with human health, especially in low-income countries. The lack of veterinary capacity in many low-income countries presents a substantial (and rising) risk to global economic and health security and causes a large ongoing human health burden in those countries. Continuing dismissive attitudes and low support to One Health approaches will reduce the effectiveness of other efforts [6]”.

In addition, the definition of the concept, adopted by the institution, underlined the economic dimension of the problem:

“One Health is a framework for enhanced collaboration in areas of common interests (intersections), with initial concentration on zoonotic diseases that will reduce risk, improve public health globally and support poverty alleviation and economic growth in developing countries. This concept involves a better way to deal with risks at the animal-human-environment interfaces [6]”.

The last event that concludes our analysis of the process of appropriation and redefinition of the *One Health* concept was the 71st United Nations General Assembly, which in 2016 was dedicated to the problem of antibiotic resistance. The resolution adopted by the members denounces as the cause of the problem inappropriate use in five sectors: public health, animal health, food, agriculture, and aquaculture. This makes it possible to observe in particular the growing awareness of the consequences of antibiotic use in aquaculture, a sector that has only recently been taken into account. In addition, the resolution qualifies the antimicrobial resistance problem as the risk that would urgently need to be addressed with the greatest attention at the global level:

“Within the broader context of antimicrobial resistance, resistance to antibiotics, which are not like other medicines, including medicines for the treatment of tuberculosis, is the greatest and most urgent global risk, requiring increased attention and coherence at the international, national and regional levels [57]”.

The need for a *One Health* approach is highlighted by emphasizing the social and economic consequences in public health, as the following excerpt shows:

“Without an effective One Health approach and other multisector cooperation and actions, antimicrobial resistance is projected to cause millions of deaths worldwide, with massive social, economic and global public health repercussions [57]”.

#### Adoption of the *One Health* approach in France and the United States

Concerning these two countries, the appropriation of the *One Health* approach by health and political actors was part of the emergence of these international events. In France, the need for a *One Health* approach has been recognized by health and political actors since 2015. This includes the report led by Dr. Jean Carlet at the request of the former Minister of Health, Marisol Touraine [11]. Structured along four axes, the report recommends: to deepen the search for new products against resistant bacteria, to better monitor the evolution of the phenomenon through shared indicators, to improve the use of antibiotics, and to raise awareness among the population about their proper use. This report highlights the need for an international *One Health* approach, while emphasizing its triple dimension that connects people, animals, and the environment:

“Only a ‘One Health’ approach, which does not separate humans from their environment (animal, food, soil, water, etc.) and allows for optimal disciplinary synergies, is likely to develop new ways of combating the emergence and spread of antibiotic resistance and to control its consequences. This approach requires a continuum between fundamental, translational, clinical, epidemiological and public health research (including the economic dimension) [11]”.

When the report was published, the need for the *One Health* approach was stressed by Marisol Touraine, whose speech at the reception of the report also highlighted collaboration with agricultural stakeholders: “it was by pursuing the same objective with Stéphane le Foll (Minister of Agriculture) that we brought health and agricultural issues together in line with the “*One Health*” initiative, which is now a reference” [49]. However, it is important to mention that, while this recognition is based on models borrowed from Scandinavian countries, notably Denmark, it was preceded by the work of Jean Yves Madec whose publications had acknowledged and emphasized the need for a *One Health* approach in regard to antibiotic resistance since 2012 [18].

The importance of the report by Jean Carlet is the creation of the first inter-ministerial committee dedicated to antibiotic resistance under the direction of Professor Christian Brun-Buisson, whose work led to the announcement in 2016, at the inter-ministerial symposium held annually since 2013, of the first roadmap divided into thirteen inter-ministerial measures and 40 actions to combat antimicrobial resistance. Based on the approaches and recommendations of international agencies, the roadmap sets out the desired coordination between the human, animal health, and environment sectors: “the 2016 end of the Antibiotic Alert Plan and the EcoAntibio Plan represents an opportunity to put in place a coordinated “*One Health*” plan to control antibiotic resistance, while respecting the specific challenges of human and animal health and the environment”. Importantly, evaluating the results of the national plans preceding 2016, whether the three national human health plans or the first EcoAntibio plan, highlighted the need for intersectoral coordination. As the second EcoAntibio plan underlined, “the loss of efficacy of antibiotics impacts human health, animal health, and ecosystem health, as these health factors are interconnected and form a whole. This is why the fight against antimicrobial resistance is a challenge to be met under a “One Health approach”.

However, despite these successive acknowledgements of the problem, it is important to remember that the various national plans in France continue to be implemented under the direction of the two ministries, respectively, that in charge of human health and that in charge of agriculture. It seems that these aspects reveal the difficulties in designing and implementing a governance model, as advocated by the *One Health* approach, in other words, a comprehensive plan.

In the United States, government recognition of links between the sectors concerned with antimicrobial resistance led to the establishment in 2015 of the first national plan under the Obama Administration, while emphasizing the need for a *One Health* approach. As the configuration and publicization process of the antibiotic resistance problem has been unique here, we recall the role played by non-governmental organizations in putting the issue on the political agenda and recognizing the need for a *One Health* approach [4]. However, as the plan points out, the approach is translated in terms of integrated bacterial resistance surveillance measures by sharing data from surveillance systems developed in different sectors (human, animal, food): “detecting and controlling antibiotic-resistance requires the adoption of a “*One Health*” approach to disease surveillance that recognizes that resistance can arise in humans, animals, and the environment”.

The brief analysis of these two different cases of national adoptions of the *One Health* approach as a solution to the antibiotic resistance problem is related to the previous questions linked to the global/international frameworks or models and their translation into national models of governance. For instance, in France, following the European ban on growth promoters (2006), the different frameworks of the issue and the recognition of the need for a *One Health* approach, several national plans have continued to be implemented separately by the ministry of health and by the ministry of agriculture. As far as the United States is concerned, the national plan launched in 2015 by the former President represents government recognition of the approach needed, despite the long history and work done by the scientific community and several NGOs in order to obtain the ban on growth promoters (applied end 2016) [4].

## Discussion: the “Antibiotic resistance – One Health” action program in the light of the descriptive/normative dichotomy of the concept

To summarize our previous observations, looking at antibiotic resistance through the *One Health* approach reveals several important aspects: firstly, it shows the political recognition of links between the fields of human and animal health, food, and the environment; secondly, it highlights two phases of re-qualification of the problem, one linked to the recognition of health actors, and the other linked to that of global political actors. At the same time, the appropriation of the concept by these actors contributes to redefinition of the meaning of the concept, and reflects actions aimed at implementing a framework for the governance of the international problem. The political movement that has been created around the problem since 2016 also highlights economic consequences and aspects, while contributing to globalization of the problem. Beyond these aspects, the analysis also suggests that competition has emerges between the agencies that take ownership of the problem in order to define the solutions, and furthermore the action programs. As previously argued, Gusfield’s analysis of public problems provides a framework for understanding the process through which different actors take ownership of problems in order to establish their definition and solutions [5]. The more actors involved, the more possible definitions and solutions. Therefore, the global agreement for a *One Health* approach needed to be translated into a plurality of solutions.

In the first part of this paper, we showed how the *One Health* concept has its origin in the former *One Medicine* concept, conceived following the emergence of zoonoses, as a call for the unification of the two medicines, human and animal. We then highlighted that the recognition of the links that these two fields maintain with the environment was associated with mobilization of an environmentalist non-governmental organization, following the rise of the problem of global warming and the emergence of epidemics and epizootics. The appropriation of the concept by international health agencies in the light of pandemics, while recognizing the economic dimensions, and also those related to food production, led to a desire to transform the concept into a model of global management and governance. This raised the question of applying a global model at the national level. Finally, an important feature of the concept’s path is that it circulated under several formulas, showing that its own signification evolved depending on the context and the actors taking ownership.

Applied to the antibiotic resistance problem, the *One Health* approach helps in stabilizing the formula as a symbol of recognition of the links between human and animal health, the environment, and food. In other words, the *One Health* formula is becoming the name of an international governing model for the problem to the detriment of the “*One Medicine”*, “*One World, One Health”*, “*One World, One Medicine”* formulas. The actors that seem to have played an important role in this process are the three international health agencies (WHO, OIE, FAO) whose discourses appear to be the international model to follow. However, when looking more closely at their declarations, the global recognition and acceptance of the *One Health* approach based on collaborations seems to be contested by different definitions of the problem. In addition to this, we should also highlight the tensions related to the need to apply a global framework to the national level.

Nonetheless, the appropriation of the problem by the plurality of international health and political actors has led to the emergence of the adjacent pair “*Antibiotic resistance – One Health*”, whose action programs underline the need for collaboration between the different sectors concerned, also taking into account the multiple dimensions revealed by these actors. While the stabilization of the *One Health* formula follows a long process, its discursive circulation includes differences in the definition of its meaning. As we can see, its definition was not quite the same at different times when the concept was applied to antibiotic resistance. This differed according to the actor who was involved in the problem. This definition may also change depending on the production of scientific knowledge proving the links between the plurality of domains. Still, understood as such, the action program includes requests for solutions that cover several aspects. As a reminder, the appropriation of the concept by the pharmaceutical industry resulted in highlighting the need for funding research into new treatments, diagnostic tools, prevention, and alternatives. On the other hand, the adoption of the approach by international health agencies underlined the awareness and recognition of sectors in agriculture, food safety, and finance, as well as the societal dimension that increasingly emphasized the need to educate consumers. Finally, the political recognition of the plurality of sectors that antimicrobial resistance links also demonstrated a phase of re-qualification of the antibiotic resistance problem. However, in implementing the approach, at least until 2016, national approaches faced difficulties and took different directions. Nevertheless, it is still important to highlight two essential aspects: on the one hand, the role played by international health agencies in the process of stabilizing the formula, reinforced by their expert position status. Their acknowledgement and “adoption” of the *One Health* approach as a framework to the antibiotic resistance problem signals the beginning of the implementation of *One Health* as these agencies are the authority in the concept’s recognition at the international level. On the other hand, the low media coverage in the national press raises questions about communication with journalists, as well as training needs on topics related to global scientific and societal problems. While on the international scene, the name seems to be an integral part of the discourse on antibiotic resistance and no one disputes the need for a *One Health* approach, both its implementation and societal publicity face difficulties at different levels (technical, methodological, national and international public resources, etc.). For example, public understanding of antibiotic use in human health and the need to follow a full course of antibiotics does not require the same tools nor the same resources when compared with the need to rethink livestock systems that would help reduce antibiotic use, nor the same action in terms of developing alternatives to antibiotics to strengthen animal immunity systems. If these very same questions could be raised at an international level, when addressed in different national contexts, the solutions would likely be adapted to specific social, political, and economic contexts.

We have therefore shown the path through which the *One Medicine* research concept evolved into the *One Health* international political governance, passing through several redefinitions according to the categories of actors taking its ownership. Since 2014, international health agencies (WHO, OIE, FAO) have been taking ownership of the concept in the context of antibiotic resistance and several collaborative or individual documents such as reports, guides, and resolutions have been published. We have pointed out recognition of the links between the fields of human and animal health, food and the environment, as well as the reframing of the problem of antibiotic resistance, in connection with recognition by global health and political actors. At the same time, the appropriation of the concept by these actors has contributed to the redefinition of the meaning of the concept, and reflects actions aimed at implementing a governing model of an international problem. Also, the application of the concept to antibiotic resistance contributes to a stabilization of the formula as a symbol of the recognition of the links between the sectors.

The end of this last section aims at questioning the implicit question of this action program resulting from looking at the antibiotic resistance problem through *One Health*: the larger question in regard to its applicability, question linked to the nature of the documents produced by the international health agencies. We noted that the three international health agencies took the lead in providing a governance model to be followed, as well as the fact that even though WHO and OIE agree on the need for collaboration and global approaches, their specific definitions of the problem do not coincide. Even though the reference international health actors involved in governing the antibiotic resistance problem agree on the need for a *One Health* approach, there are difficulties related to schemes and patterns to apply or indicators to develop at national levels in order to remain consistent with what is considered at the international level. The status of these international health agencies and the documents they have been producing are also important. For instance, social sciences researchers have long studied the characteristics of “reports” pointing out their functioning as tools for rational decision support with a dual aim: to inform and describe, as well as to evaluate and prescribe [51]. Since their documents are guides and recommendations, it seems that the *One Health* concept is rather seen as a descriptive concept, and not as a normative one. These documents contribute to producing operational knowledge, especially since the agencies’ expert status legitimates the knowledge produced. However, in terms of performativity, these documents are not regulations but guides and recommendations. For example, the *One Health* development trajectory can be compared with other concepts related to the antibiotic resistance issue such as *antibiotic-free*. Its application in practice seems to be established by norms and regulations regarding the use of antibiotics in livestock, differently from one country to another. This consideration implies that this second concept is a normative one, while *One Health* remains still descriptive. Furthermore, the difficulties in establishing a *One Health* model can be closely linked to a lack of specific regulations. Regarding antibiotic use in livestock, countries such as France and the United States have benefited from a transition period between massive use and the ban on growth promoters. What models following the *One Health* approach are then to be considered and developed for countries where regulations have only recently been implemented, acknowledging the different economic and social contexts?

Clearly, the international health agencies work contributes to producing operational knowledge in regard to the global problem of the antibiotic resistance. To quote one last time Joseph Gusfiel: “concepts (…) develop in connection with problems of morals, politics, ethics and human choice and enable us to pick out and assess those activities which have relevance for our interests, purposes and sentiments. For this reason, concepts are best understood by seeing how they are used, by examining their historical developments, by showing their contrasting concepts and even by criticizing theirs claims and uses” [30]. The question that remains is linked to national approaches to follow and implement under the *One Health* approach. Moreover, another important dimension concerns the responsibilities and implications that arise from the way in which public authorities, industry and the public appropriate a concept. As the *One Health* approach seems to be largely established within health actors’ rhetoric, studies remain to be carried out on the public’s understanding. In this sense, the participation of the public in the implementation of both public policies and solutions developed by industry highlights the need to mobilize society in the search for solutions. In this respect, from a communication point of view, media participation in this dynamic of concept constitution and problem publicization is to be analyzed, in particular the role they play in the process of publicizing and establishing the meaning of the names of the problem and the formulas defining its causes and solutions.

## Appendix

**Table A1:** Health and political actor documents

Date	Author	Title	Reference
2014	WHO	Antimicrobial resistance. Global report on surveillance	[54]
2015	WHO	Global action plan on antimicrobial resistance	[55]
2015	ECDC/EFSA/EMA	First joint report on the integrated analysis of the consumption of antimicrobial agents and occurrence of antimicrobial resistance in bacteria from humans and food-producing animals	[19]
2016	ECDC/EFSA/EMA	The European Union summary report on antimicrobial resistance in zoonotic and indicator bacteria from humans, animals and food in 2014	[20]
2016	Pharmaceutical Industry	Declaration by the Pharmaceutical, Biotechnology and Diagnostics Industries on Combating Antimicrobial Resistance	[58]
2016	G7	G7 Ise-Shima Leaders’ Declaration	[31]
2016	G7	G7 Ise-Shima Vision for Global Health	[32]
2016	G20	G20 Leaders’ Communique	[33]
2016	World Bank	Drug-Resistant Infections: A Threat to Our Economic Future	[6]
2016	UN	Resolution adopted by the General Assembly on 5 October 2016	[57]
2016	FAO	The FAO action plan on antimicrobial resistance 2016–2020	[21]
2016	OIE	Resolution 36: Combating Antimicrobial Resistance through a One Health Approach: Actions and OIE Strategy	[53]
2016	OIE	The OIE Strategy on Antimicrobial Resistance and the Prudent Use of Antimicrobials	[52]
France			
2006	JMA/DGS	Assessment of the plan to preserve the efficacy of antibiotics 2001–2005 (*Bilan du plan pour préserver l'efficacité des antibiotiques 2001-2005*)	[35]
2007	JMA/DGS	Assessment of the plan to preserve the efficacy of antibiotics 2007–2010 (*Bilan du plan pour préserver l'efficacité des antibiotiques 2007-2010*)	[36]
2011	Ministry of Health	National alert plan on antibiotics 2011–2016 (*Plan national d'alerte sur les antibiotiques 2011-2016*)	[50]
2015	Jean Carlet	Together, let’s save antibiotics (*Tous ensemble sauvons les antibiotiques*)	[11]
2016	Interministerial Committee	Roadmap on antibiotic resistance (*Feuille de route antibiorésistance*)	[17]
2016	Ministry of Agriculture	2012–2016 Ecoantiobio plan – assessment and recommendations (*Le plan écoantibio 2012-2016 évaluation et recommandations*)	[47]
2016	Ministry of Agriculture	Ecoantibio2 plan – National plan on the reduction of risks related to antibiotic resistance in veterinary medicine (*Ecoantibio2 Plan national de réduction des risques d'antibiorésistance en médecine vétérinaire*)	[46]
United States			
2012	Food and Drug Administration	Guidance 209	[24]
2013	Food and Drug Administration	Guidance 213	[25]
2013	Food and Drug Administration	Veterinary Feed Directive	[26]
2013	Center for Disease Control and Prevention	Antibiotic Resistance Threats in the United States	[14]
2015	White House	National Action Plan for Combating Antibiotic-Resistant Bacteria	[60]
